# Promotion of colorectal cancer cell death by ezetimibe *via* mTOR signaling-dependent mitochondrial dysfunction

**DOI:** 10.3389/fphar.2023.1081980

**Published:** 2023-02-07

**Authors:** Yuanyuan Zheng, Wenjuan Yang, Yewei Jia, Jie Ji, Liwei Wu, Jiao Feng, Yan Li, Ziqi Cheng, Jie Zhang, Jingjing Li, Weiqi Dai, Xuanfu Xu, Jianye Wu, Yingqun Zhou, Chuanyong Guo

**Affiliations:** ^1^ Department of Gastroenterology, Shanghai Tenth People's Hospital, Tongji University School of Medicine, Shanghai, China; ^2^ Department of Emergency, Shanghai Tenth People's Hospital, School of Medicine, Shanghai, China; ^3^ Department of Internal Medicine 3, Friedrich-Alexander-University Erlangen-Nürnberg (FAU) and Universitätsklinikum Erlangen, Erlangen, Germany; ^4^ Department of Gastroenterology, Shidong Hospital, Shanghai, China; ^5^ Department of Gastroenterology, Putuo People’s Hospital, Tongji University, Shanghai, China

**Keywords:** colorectal cancer, cell death, ezetimibe, MTOR signaling, mitochondrial dysfunction

## Abstract

**Introduction:** Colorectal cancer (CRC) is the fourth most common cancer worldwide, with high morbidity and mortality rates. In recent years, high-fat diet has been shown to increase CRC morbidity, highlighting the possibility of the application of hypolipidemic drugs for CRC treatment. In this study, we preliminarily evaluated the effects and mechnisms of ezetimibe against CRC through the blockage of lipid absorption in small intesine.

**Methods:** In this study, CRC cell proliferation, invasion, apoptosis, and autophagy were evaluated using cellular and molecular assays. Fluorescent microscopy, and a flow cytometric assay were used to assess mitochondrial activity *in vitro*. A subcutaneous xenograft mouse model was used to evaluate the effects of ezetimibe *in vivo*.

**Results:** We found that ezetimibe inhibited CRC cell proliferation, and migration, and facilitated autophage-associated apoptosis in HCT116 and Caco2 cells. Ezetimibe-induced mitochondrial dysfunction in CRC cells was found to be correlated with mTOR signaling activity.

**Discussion:** Ezetimibe exhibits effects against CRC through the promotion of cancer cell death *via* mTOR signaling-dependent mitochondrial dysfunction, highlighting its potential value in CRC therapy.

## Introduction

Colorectal cancer (CRC), is one of the most frequently occurring digestive malignancies worldwide and accounts for approximately 9.4% and 10.1% of all cancers in man and woman (Boyle and Langman, 2000; Benson et al., 2018). Environmental and hereditary factors, such as diet, dysbacteriosis, and genetic variations, have been found to be the most significant risk factors for CRC morbidity (Boyle and Langman, 2000; Grady, 2003; Garrett, 2019; Vernia et al., 2021). The main treatment strategies for CRC include endoscopic therapy for early stage CRC and radical surgery with adjuvant systemic therapy for advanced CRC (Benson et al., 2014; Benson et al., 2018). Preventing of CRC etiology and optimizing systemic therapeutic strategies against the disease are both beneficial strategies for improving overall CRC patient survival.

Laboratory-based and clinical studies have found high dietary fat intake, as well as bile acid metabolism by gut microbiota, account for the increasing risk of CRC incidence (Boyle and Langman, 2000; Garrett, 2019; Ocvirk and O'Keefe, 2021). Previous study on correlations between CRC and cholesterol metabolism provided evidence of confirming serum cholesterol as carcinogenic factor on CRC progression, and an abnormal accumulation of formed secondary bile acids also presented promoting effects on CRC (Jacobs et al., 2012). Thus, drugs that target lipid metabolism may have potential benefits in CRC adjuvant therapy. Stain, an HMG-CoA reductase inhibitor which exerts significant effects in lowering cholesterin (TC) and low-density lipoprotein (LDL) levels, was found to exhibit protective effects against CRC by reducing the dilatation activity and enhancing the chemotherapeutic sensitivity of CRC cells (Bardou et al., 2010). In addition, studies have demonstrated the anti-tumor effects of fibrates, which function mainly in reducing LDL and triglyceride (TG) levels, against CRC and other digestive neoplasms (Chen et al., 2020; Kong et al., 2021; Li et al., 2021). Cholesterol accumulation, which exceeded the ability of liver conversion and intestinal absorption, was reported as high risk factor of CRC (Waluga et al., 2018; Gu et al., 2022). Squalene epoxidase (SQLE), a cholesterol synthesis promoting factor, has been proved to suppress the apoptosis of CRC cells and lead to poor disease outcome (Li et al., 2022). And the inhibition impacts of cholesterol on CRC cell apoptosis has also been reported *via* miR-33a-PIM3 pathway (Wang et al., 2019). As ezetimibe blocks lipid absorption in the small intesines, which is the main organ for bile acid re-absorption, it may also have potential anti-CRC effects, even though correlational approaches to prove this are lacking. Furthermore, the high toxicity of adjuvant chemotherapy may partially counteract its benefits in CRC patients, especially in older patients under combined chemotherapeutic regimens (Moth et al., 2016; Benson et al., 2018). Studies on novel candidates for adjuvant therapy against CRC, which focus on reversing damages counteracted benefits, are still being carried out. In this study, we preliminarily investigated the effects and possible mechanisms of ezetimibe against CRC.

## Materials and methods

### Medications and antibodies

Ezetimibe and MHY1485 were purchased from MedChemExpress (Shanghai, China). Anti-β-actin antibodies were purchased from Sigma-Aldrich. Anti-mTOR, anti-caspase-8, and anti-caspase-9 antibodies were obtained from Cell Signaling Technology, Inc. Anti-MMP-9 and anti-MMP-2 were purchased from Abcam. Anti-caspase-3 antibodies were purchased from Abmart. Anti-MTOR^pSer2448^, anti-Beclin-1, anti-LC3, anti-BAX and anti-Bcl-2 antibodies were obtained from ProteinTech, Inc. Other reagents and kits are listed under the corresponding methods.

### Cell culture

The two human colorectal carcinoma cell lines, HCT116 and Caco2, were obtained from the cell bank of the Chinese academy of sciences. The cell culture fluid used for cultivating HCT116 cells was the RPMI-1640 medium supplemented with 10% fetal bovine serum (FBS) and 1% penicillin-streptomycin. DMEM supplemented with 10% FBS and 1% penicillin-streptomycin was used to culture Caco2 cells. The temperature of the cell culture incubator was set at 37°C and it contained 5% CO2 and humidified air.

### Cell viability assay

HCT116 and Caco2 cells were seeded and incubated in 96-well plates. After 12 h of incubation for cell attachment, ezetimibe was added to each group (6 wells per group) at increasing concentrations (0, 1, 2, 4, 8, 16, 32, 64, and 128 μM in culture medium). Cell viability was evaluated at 24 and 48 h with Cell-Counting-Kit-8 reagent (MedChemExpress), which detected by using a microplate reader (450 nm). This assay was repeated thrice. The half maximal inhibitory concentration (IC_50_) was calculated using GraphPad Prism 9.0 (GraphPad Software).

### Scratch assay

HCT116 and Caco2 cells were seeded in 6-well plates and cultured normally for 36 h and then starved separately for 12 h. Then, scratches were made on the surface of the cells in each well using 100-μL sterile pipette tips. Subsequently, phosphate buffer saline (PBS) was used to wash off cell debris and then ezetimibe was added into each well (0, 20, 40, and 60 μM ezetimibe in the HCT116 cell culture medium; 0, 40, 60, and 80 μM ezetimibe in the Caco2 cell culture medium). The wound area was photographed using an optical microscope at 0, 24, and 48 h. ImageJ 1.8.0 (National Institutes of Health) was used to measure the wound area.

### Clone formation assay

CRC cells were seeded and cultured in 6-well plates and differently treated with ezetimibe refer to the concentrations in scratch assay for 48 h. Then, the cells were cultured for another 2 weeks in normal culture medium. A 0.1% crystal violet solution was used for clone staining after 30 min of fixation using 4% paraformaldehyde. ImageJ 1.8.0 was used to count cell colonies.

### Fluorescence microscopic assay

#### Apoptosis detection using Hoechst33258

HCT116 and Caco2 cells were seeded and cultured in 96-well plates. After cell adherence, ezetimibe was added to each well following to the concentrations in scratch assay. Then, the cells were stained with Hoechst33258 in lucifuge after fixation using 4% paraformaldehyde. The nuclear morphology of colon cells was captured by fluorescence microscopy after the cells were washed with PBS.

#### Mitochondrial membrane potential measurement using JC-1

The enhanced mitochondrial membrane potential assay kit and JC-1 (Beyotime Institute of Biotechnology) were used to assess the mitochondrial membrane potential (△Ψm) of ezetimibe-treated CRC cells. HCT116 and Caco2 cells were seeded and treated with ezetimibe in 6-well plates with previous mentioned concentrations for 48 h. Then, the cells were incubated with JC-1 for 20 min at 37°C and washed twice with JC-1 dyeing buffer. The fluorescence intensities of JC-1 monomers (excitation wavelength: 490 nm; emission wavelength: 530 nm) and aggregates (excitation wavelength: 525 nm; emission wavelength: 590 nm) were measured by fluorescence microscopy.

#### Evaluation of intracellular reactive oxygen species (ROS) using the ROS kit

The Reactive Oxygen Species Assay Kit (Beyotime Institute of Biotechnology) was used to detect intracellular ROS-oxidized 2′-7′ DCF. CRC cells (HCT116 and Caco2) were cultured with ezetimibe followed the mentioned concentrations in stratch assay for 48 h in 6-well plates. DCFH-DA was diluted with serum-free medium and loaded onto CRC-containing wells for ROS detection. After incubating the cells for 20 min at 37°C in a cell incubator and washing them twice with serum free medium, intracellular CRC cell ROS levels were measured by fluorescence microscopy (excitation wavelength: 488 nm; emission wavelength: 525 nm).

### Flow cytometric assay

#### Evaluation of apoptosis using Annexin V-FITC/PI

The Annexin V-FITC Apoptosis Detection Kit (Beyotime Institute of Biotechnology) was used to evaluate apoptosis. HCT116 and Caco2 cells were treated with incremental concentrations of ezetimibe as previous and harvested after 48 h into flow tubes. Then, the cells were washed with PBS and dyed with Annexin V-FITC/propidium iodide (PI) for 20 min in the dark. Fluorescence signals were detected using a BD LSRFortessa™ (Becton, Dickinson and Company) device.

#### △Ψm monitoring using JC-1

HCT116 and Caco2 were treated and dyed as described for the JC-1 fluorescence microscopic assay. Then, the cells were harvested and resuspended in JC-1 dyeing buffer. Finally, fluorescence signals for JC-1 monomers and aggregates were detected using the BD LSRFortessa™ device.

#### Evaluation of intracellular ROS expression using the ROS kit

HCT116 and Caco2 cells were intervened and stained following the specifications for the fluorescence microscopic assay for ROS. The two CRC cell types were collected and subjected to flow cytometric analysis using the BD LSRFortessa™ device.

#### Bioinformatics-based exploration of ezetimibe-targeted genes and molecular mechanisms

Ezetimibe-targeted genes were searched in the Comparative Toxicogenomics Database (NC State University) and Drugbank online (OMx Personal Health Analytics, Inc.) using the key word, “ezetimibe.” String version 11.5 (ELIXIR) was used to display the corresponding protein interaction networks of the targeted genes. Then, the genes were retrieved in the Gene Expression Profiling Interactive Analysis (GEPIA) (Peking university) database for the determination of the expression levels of the genes in CRC and normal tissues. Differentially expressed targeted genes were further searched to determine gene expression at different CRC stages; in addition, these genes were retrieved in the GEPIA database for the comparison of patient overall survival between the high and low expression groups. The correlation between the expression levels of several key metabolic genes and ezetimibe-targeted genes were also searched in the GEPIA database.

#### Protein detection by western blotting

CRC cells were treated with ezetimibe as previous for 48 h, then collected and washed with PBS. The RIPA lysis buffer with PMSF and a protein phosphatase inhibitor were used to prepare the cell lysis buffer. The proteins were quantified using the Enhanced BCA Protein Assay Kit (Beyotime Institute of Biotechnology) and then heat-denatured using the loading buffer (Epizyme Biotech) at 95°C for 5 min. Sodium dodecyl sulfate polyacrylamide gel electrophoresis was used for protein separation in each group. Then, the proteins were transferred onto Polyvinylidene fluoride (PVDF) membranes (Millipore Corp.) and blocked with the Protein Free Rapid Blocking Buffer (Epizyme Biotech). Protein bands were incubated with the corresponding primary antibodies for 12 h at 4°C (anti-β-actin, anti-MTORpSer2448, anti-mTOR, anti-caspase-3, anti-caspase-8, anti-caspase-9, anti-MMP-9, anti-MMP-2, anti-p-62, anti-LC3, anti-BAX, and anti-Bcl-2 in antibody diluent, New cell & Molecular Biotech CO., Ltd., WB500D). Next, the bands were washed with Tris Buffered Saline Tween and incubated with HRP-linked secondary antibodies for 1 h. BeyoECL Moon (Beyotime Institute of Biotechnology) was used to visualize the protein bands and Amersham Imager 600 (Cytiva) was used to capture optical signals.

#### Assessment of RNA transcription through qRT-PCR analysis

Total RNA was extracted from CRC cells treated with ezetimibe following the instructions of the EZ-press RNA Purification Kit (EZBioscience) manufacturer. NanoDrop One/Oneᶜ (Thermo Fisher scientific) was used to determine the concentration and purity of the purified RNA. A total purified RNA quantity of 1 μg was obtained from each group and reverse transcribed to obtain cDNA following the instructions of the PrimeScript™ RT reagent Kit (Takara Bio) manufacturer. Forward and reverse primers were obtained from Generay Biotechnology and are listed in Table 1 qRT-PCR was performed using the Hieff UNICON^®^ qPCR SYBR Green Master Mix (Yeasen) and the amplification products were detected using the QuantStudio Dx Real-Time PCR device (Thermo Fisher scientific). β-actin gene expression was monitored as the control, and the 2^△△CT^ formula was used to calculate the relative expression levels of the evaluated genes.

**TABLE 1:** Clinical features and correlations to GLS, HIF1-α, mTOR, TNF, p53 for the 29 ezetimibe targeted genes marked by the Comparative Toxicogenomics Database and Drugbank.

Target genes	Gene expression difference between colon cancer and normal tissue	Gene expression difference between colon cancer stages	Correlation between gene expression and overall survival	Correlations between ezetimibe targeted genes and key energy metabolism indicators in colon cancer				
				GLS	HIF1-α	mTOR	TNF	P53
NPC1L1	—	F value 0.488 pr (>F) 0.691	Logrank p 0.44	p0.95 R0.0036 **	p0.31 R0.058	p0.29 R-0.06	p0.5 R-0.038	p0.33 R0.054
HMGCR	*	F value 0.987 pr (>F) 0.399	Logrank p 0.097	p0.37 R0.051	p0.0085 R0.15 **	p3.1e-7 R0.28 ***	p0.85 R-0.011	p0.28 R0.062
APOA1	—	F value 0.406 pr (>F) 0.749	Logrank p 0.24	p0.0013 R0.18 **	p0.54 R-0.034	p0.67 R-0.024	p0.79 R-0.015	p0.19 R-0.074
LDLR	*	F value 1.12 pr (>F) 0.34	Logrank p 0.3	p0.27 R0.063	p9.1e-7 R0.27 ***	p0.00035 R0.2 ***	p0.059 R0.11	p0.097 R0.094
SREBF2	—	F value 0.816 pr (>F) 0.487	Logrank p 0.27	p0.13 R0.086	p0.0061R0.15 **	p8.9e-16 R0.43 ***	p0.011 R0.14 *	p0.33 R0.055
ABCB11	—	F value 1.53 pr (>F) 0.207	Logrank p 0.94	p0.029 R-0.12 *	p0.61 R-0.029	p0.44 R-0.044	p0.9 R-0.0068	p0.0025 R-0.17 **
CRP	-	F value 1.03 pr (>F) 0.38	-	p0.55 R-0.034	p0.42 R-0.045	p0.11 R-0.089	p0.91 R-0.0064	p0.059 R0.11
SREBF1	—	F value 0.355 pr (>F) 0.785	Logrank p 0.29	p0.016 R-0.14 *	p4.2e-6 R0.26 ***	p1.3e-5 R0.24 ***	p0.42 R0.045	p1.1e-6 R0.27 ***
TNF	—	F value 0.739 pr (>F) 0.53	Logrank p 0.22	p0.71 R0.021	p0.001 R0.18 **	p0.057 R0.11	-	p0.96 R0.0027
ABCA1	*	F value 0.687 pr (>F) 0.561	Logrank p 0.23	p0.098 R0.093	p<0.001 R0.54 ***	p5.3e-7 R0.28 ***	p2.3e-5 R0.24 ***	p0.025 R-0.13 *
NR1H2	—	F value 1.55 pr (>F) 0.201	Logrank p 0.26	p0.012 R-0.14 *	p0.0051 R0.16 **	p0.07 R0.1	p0.00017 R0.21 ***	p0.065 R -0.1
PCSK9	*	F value 0.6 pr (>F) 0.615	Logrank p 0.67	p0.67 R-0.024	p6.4e-5 R0.22 ***	p2.4e-5 R0.24 ***	p0.46 R0.042	p4.2e-5 R0.23 ***
PGR	*	F value 3.64 pr (>F) 0.0133 *	Logrank p 0.021 *	p0.14 R-0.082	p0.0037 R0.16 **	p0.06 R0.11	p0.31 R0.057	p0.0034 R-0.16 **
PLA2G7	*	F value 1.57 pr (>F) 0.198	Logrank p 0.85	p0.0094 R0.15 **	p<0.001 R0.45 ***	p0.00026 R0.2 ***	p2.4e-9 R0.33 ***	p0.64 R-0.026
RARG	-	F value 1.49 pr (>F) 0.218	Logrank p 0.55	p0.064 R0.1	p0.17 R0.077	p0.00024 R0.21 ***	p0.64 R0.026	p0.61 R0.029
SCARB1	*	F value 0.917 pr (>F) 0.433	Logrank p 0.33	p0.011 R0.14 *	p0.015 R-0.14 *	p0.27 R0.062	p0.45 R-0.043	p0.003 R0.17 **
UGT1A1	—	F value 1.57 pr (>F) 0.197	Logrank p 0.91	p0.68 R0.023	p0.42 R-0.046	p0.35 R-0.063	p0.64 R-0.026	p0.011 R-0.14 *
UGT1A3	-	F value 2.61 pr (>F) 0.0519	Logrank p 0.26	p0.0011 R0.18 **	p0.14 R-0.083	p0.26 R-0.064	p0.57 R-0.032	p0.091 R-0.095
UGT2B15	-	F value 0.058 pr (>F) 0.982	Logrank p 0.027 *	p0.00038 R-0.2 ***	p0.63 R0.027	p0.88 R-0.0087	p0.9 R0.0072	p0.00039 R-0.2 ***
UGT2B7	—	F value 2.48 pr (>F) 0.0681	Logrank p 0.041 *	p0.61 R0.029	p0.93 R-0.0046	p0.82 R-0.013	p0.53 R-0.036	p0.85 R0.011
SOAT1	—	F value 0.45 pr (>F) 0.717	Logrank p 0.69	p0.039 R0.12 *	p8.9e-16 R0.43 ***	p6.8e-6 R0.25 ***	p0.0022 R0.17 **	p0.64 R0.027
ANPEP	—	F value 0.127 pr (>F) 0.944	Logrank p 0.21	p0.42 R0.046 *	p0.73 R-0.02	p0.74 R-0.019	p0.93 R-0.0052	p0.0023 R-0.17 **
CYP3A4	—	F value 0.615 pr (>F) 0.606	Logrank p 0.25	p0.0022 R0.17 **	p0.5 R-0.038	p0.51 R-0.037	p0.82 R-0.013	p0.12 R-0.087
CYP2C8	—	F value 0.324 pr (>F) 0.808	Logrank p 0.32	p0.049 R0.11 *	p0.71 R-0.021	p0.87 R-0.0089	p0.96 R-0.0031	p0.096 R-0.094
ABCC2	—	F value 0.912 pr (>F) 0.436	Logrank p 0.28	p2.8e-8 R0.31 ***	p0.054 R-0.11	p0.22 R0.069	p0.21 R-0.071	p0.34 R0.054
ABCB1	—	F value 1.54 pr (>F) 0.204	Logrank p 0.42	p0.0036 R0.16 **	p0.062 R-0.11	p0.84 R0.011	p0.98 R-0.0012	p0.17 R-0.078
ABCC3	*	F value 4.67 pr (>F) 0.00338 **	Logrank p 0.62	p0.0056 R-0.16 **	p0.24 R-0.067	p1e-6 R0.27 ***	p0.97 R-0.0019	p0.095 R-0.094
SLCO1B1	—	F value 1.59 pr (>F) 0.193	Logrank p 0.26	p0.37 R-0.051	p0.5 R0.0038	p0.32 R-0.056	p0.5 R-0.038	p0.014 R0.14 *
ABCG2	—	F value 0.831 pr (>F) 0.478	Logrank p 0.7	p0.96 R-0.0028	p0.32 R-0.056	p0.19 R-0.073	p0.3 R0.058	p7.1e-5 R-0.22 ***

#### Animals

Male BALB/c-nu mice aged 4–6-weeks were purchased from Shanghai SLAC Laboratory Animal CO. Ltd. (Organization code no. 74616122-2) and raised in Shanghai Rat&Mouse Biotech Co., Ltd. (Organization code no. 59814249-1). HCT116 cells were injected into the right subcutaneous part of each mouse before the mice were randomly divided into two groups (*n* = 5). Ezetimibe, suspended in corn oil, was intragastrically administered to each mouse in the treatment group at a daily dose of 50 mg/Kg. In addition, mice in the control group were intragastrically administered the same quantity of corn oil once daily. Then, the mice were anesthetized and sacrificed after 3 weeks of intervention. The weight of each mouse and the size of the subcutaneous tumor were recorded. Next, mouse hepatic, nephric, and xenograft tumor tissues were collected. The tissues were subjected to Hematoxylin and Eosin (HE) and immunohistochemical staining, and observed under a light microscope; next, the tissues were fixed with 4% araformaldehyde and paraffin-embedded. Tissues for transmission electron microscopic analysis were fixed with the 2.5% glutaraldehyde fixing solution (SenBeiJia Biological Technology Co., Ltd.).

#### Statistical analysis

SPSS 24.0 (IBM) was used to calculate statistical differences between groups. Min-max (MM) normalization was applied for data before quantitative analysis (Dinç et al., 2014). One-way analysis of variance was used for multiple group analyses, and Student’s *t*-test was used for comparison between two groups. Values of *p* value < 0.05 were considered statistically significant. Experiments were carried out in triplicate.

## Results

### Ezetimibe promotes CRC cell death

**FIGURE 1:**
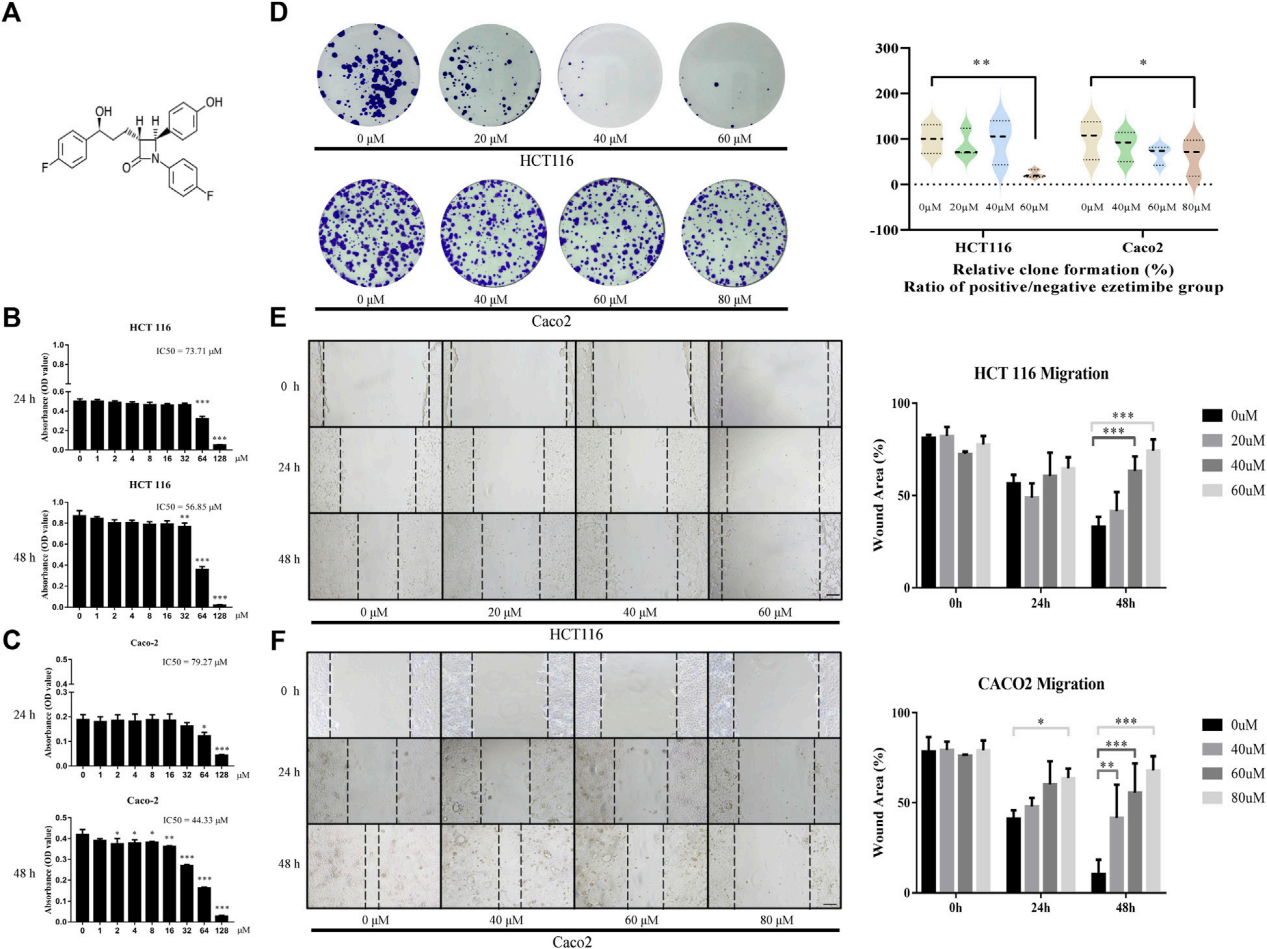
Ezetimibe suppresses the CRC cell activity by decreasing its proliferation and restraining its migration. **(A)** Chemical structure of ezetimibe. **(B)** Effects of ezetimibe on the viability of HCT116 at 24 and 48 h detected by CCK-8 assay. **(C)** Effects of ezetimibe on the viability of Caco2 at 24 and 48 h detected by CCK-8 assay. **(D)** The variations of clone formation for HCT116 and Caco2 cells under gradient ezetimibe treatment at 48 h. **(E)** The wound healing capacity for HCT116 under gradient ezetimibe treatment at 0, 24 and 48 h. Scale bar, 100 μM. **(F)** The wound healing capacity for Caco2 under gradient ezetimibe treatment at 0, 24 and 48 h. Scale bar, 100 μM *p < 0.05, **p < 0.01, ***p < 0.001.

**FIGURE 2:**
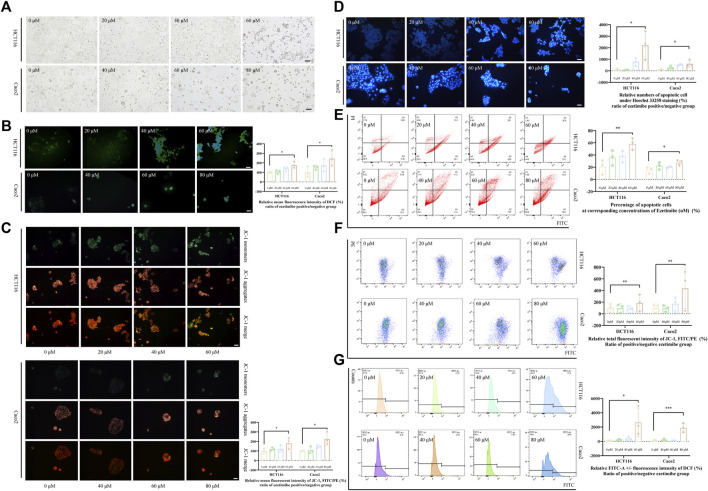
Ezetimibe promotes the CRC cell death with mitochondrial dysfunction. **(A)** Morphological changes of HCT116 and Caco2 cells under gradient ezetimibe treatment at 48 h. **(B)** Fluorescence intensity of DCF in gradient ezetimibe treated HCT116 and Caco2 cells with microscopic observation. Scale bar, 100 μM. **(C)** Fluorescence intensity of JC-1 in gradient ezetimibe treated HCT116 and Caco2 cells with microscopic observation. Scale bar, 100 μM. **(D)** Apoptosis of HCT116 and Caco2 cells under gradient ezetimibe treatment with hoechst33258 staining. Scale bar, 100 μM. **(E)** Apoptosis of HCT116 and Caco2 cells under gradient ezetimibe treatment with Annexin V-FITC/PI staining flow cytometry. **(F)** Fluorescence intensity of JC-1 in gradient ezetimibe treated HCT116 and Caco2 cells with flow cytometry. **(G)** Fluorescence intensity of DCF in gradient ezetimibe treated HCT116 and Caco2 cells with flow cytometry. *p < 0.05, **p < 0.01, ***p < 0.001.

Ezetimibe suppressed CRC cell viability by decreasing their proliferation, inhibiting their migration, and facilitating their apoptosis. The CCK-8 assay showed that ezetimibe significantly inhibited HCT116 cell proliferation (Figure 1B), as well as Caco2 cell proliferation (Figure 1C), in a dose-dependent manner; the concentration gradients for subsequent experiments were strictly determined based on the ezetimibe IC_50_ values obtained in the two CRC cell lines. Optical microscopic analysis revealed a significant increase in growth arrest-associated cell death in the CRC cell lines with increase in ezetimibe concentrations at 48 h (Figure 2A). The findings of the clone formation assay also demonstrated the cytostatic action of ezetimibe on these CRC cell lines (Figure 1D). The scratch assay showed that ezetimibe attenuated CRC cell migration (Figures 1E, F). Hoechst 33258 staining and flow cytometry (Annexin V-FITC/PI staining) were performed to evaluate the CRC cell apoptosis under ezetimibe treatment. The proportion of apoptotic cells with condensed and fragmented nuclei significantly increased with increase in ezetimibe effective concentrations at 48 h (Figure 2D). Annexin V-FITC/PI staining also showed a significant increase in CRC cell apoptosis (Figure 2E).

### Ezetimibe promotes CRC cell death by inducing mitochondrial damage in these cancerous cells

The effective lethal concentration of ezetimibe was found to decrease the △Ψm of CRC cells and increase ROS levels in these cells. The decrease in △Ψm is revealed by an upregulation of the JC-1 monomer/aggregate ratio, which was observed when effective ezetimibe concentrations in the treatment and control group were compared. HCT116 and Caco2 cells showed a significant decrease in △Ψm both in the fluoresce microscopic analysis (Figure 2C) and the flow cytometric assay (Figure 2F) when effective concentrations in the treatment and control group were compared. Intracellular ROS levels, as determined using 2′,7′-Dichlorodihydrofluorescein diacetate (DCFH-DA), were found to increase in the ezetimibe treatment groups as compared to the control group in both HCT116 and Caco2 cells with fluorescence microscopy and flow cytometry (Figure 2B, G). Relative FITC-A positive against negative fluorescence intensity of dichlorofluorescein (DCF) was calculated between the ezetimibe treated and control groups in a fold range manner for the flow cytometric assay.

### Ezetimibe induced mitochondrial dysfunction in colonic cancerous cells by inhibiting the mTOR signaling pathway

**FIGURE 3:**
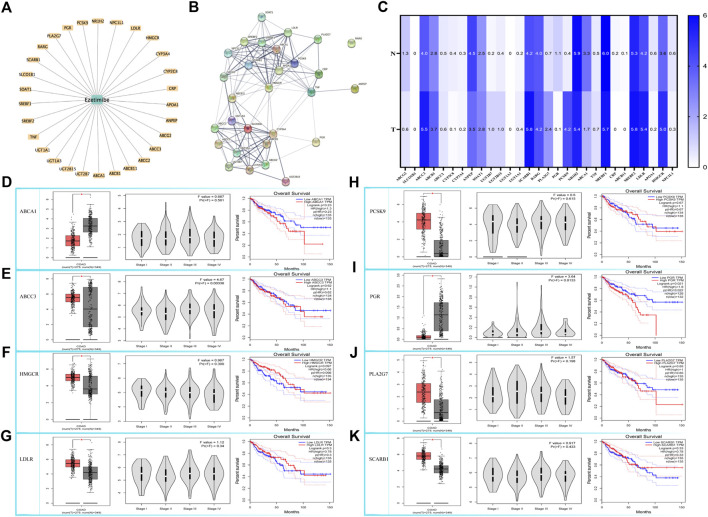
Bioinformatic based explorations of ezetimibe targeted genes and clinical correlations for CRC related ones. **(A)** The Comparative Toxicogenomics Database and Drugbank online based retrieval of targeted genes for ezetimibe. **(B)** Protein string for the retrieved targeted genes. **(C)** The Gene Expression Profiling Interactive Analysis (GEPIA) based expression fold changes between tumor and normal tissue in CRC patients. **(D–K)** GEPIA based CRC related expressed genes (ABCA1, ABCC3, HMGCR, LDLR, PCSK9, PGR, PLA2G7, SCARB1) and the correlations with their expressions to CRC stages, and to CRC overall survival. *p < 0.05.

Our bioinformatics analysis revealed a significant correlation between ezetimibe and mTOR signaling in CRC cells. A total of 29 ezetimibe-targeted genes were marked based on data obtained from the Comparative Toxicogenomics Database and Drugbank (Figure 3A); these genes mainly clustered into groups of key elements under both glycometabolism and lipometabolism (Figure 3B). The expression levels of eight out of the 29 targeted genes, including *ABCA1*, *ABCC3*, *HMGCR*, *LDLR*, *PCSK9*, *PGR*, *PLA2G7*, *and SCARB1*, were significantly different between colonic tumor tissues and normal tissues (Figure 3C). The expression levels of *PGR* and *ABCC3* were significantly different between the colorectal cancer stages (Table 2; Figure 3E). In addition, *PGR*, as well as *UGT2B15* and *UGT2B7*, which were the non-tumor-related genes identified among the 29 targeted genes, were found to be related to overall survival in CRC patients (Table 2; Figure 3I). Among the eight tumor-related genes, *ABCA1* and *PLA2G7* were found to be significantly related to TNF (Table 2). Three of the eight colonic tumor-related genes, *ABCA1*, *PLA2G7*, and *SCARB1*, were significantly correlated with GLS (Table 2), the main enzyme for glutamate metabolism. *ABCA*, *PCSK9*, *PGR*, and *SCARB1* were found to be significantly associated with p53 (Table 2). As concerns the mTOR signaling pathway, six of the eight targeted genes were found to be significantly associated with mTOR, and seven of these genes were significantly associated with downstream HIF1-α: *HMGCR*, *LDLR*, *ABCA1*, *PCSK9*, *PLA2G7*, and *ABCC3* for mTOR*,* and *HMGCR*, *LDLR*, *ABCA1*, *PCSK9*, *PGR*, *PLA2G7*, and *SCARB1* for HIF1-α (Table 2).

**TABLE 2:** Forward and reverse primers of β-actin, mTOR, Beclin-1, LC3 I, LC3 II, BAX and BCL2 for qRT-PCR analysis.

Genes		Primer sequence	Base number
β-actin	Forward	CAT​GTA​CGT​TGC​TAT​CCA​GGC	21
	Reverse	CTC​CTT​AAT​GTC​ACG​CAC​GAT	21
mTOR	Forward	TCC​GAG​AGA​TGA​GTC​AAG​AGG	21
	Reverse	CAC​CTT​CCA​CTC​CTA​TGA​GGC	21
Beclin-1	Forward	ACC​TCA​GCC​GAA​GAC​TGA​AG	20
	Reverse	AAC​AGC​GTT​TGT​AGT​TCT​GAC​A	22
LC3 I	Forward	AAC​ATG​AGC​GAG​TTG​GTC​AAG	21
	Reverse	GCT​CGT​AGA​TGT​CCG​CGA​T	19
LC3 II	Forward	GAT​GTC​CGA​CTT​ATT​CGA​GAG​C	22
	Reverse	TTG​AGC​TGT​AAG​CGC​CTT​CTA	21
BAX	Forward	CCC​GAG​AGG​TCT​TTT​TCC​GAG	21
	Reverse	CCA​GCC​CAT​GAT​GGT​TCT​GAT	21
BCL2	Forward	GGT​GGG​GTC​ATG​TGT​GTG​G	19
	Reverse	CGG​TTC​AGG​TAC​TCA​GTC​ATC​C	22

**FIGURE 4:**
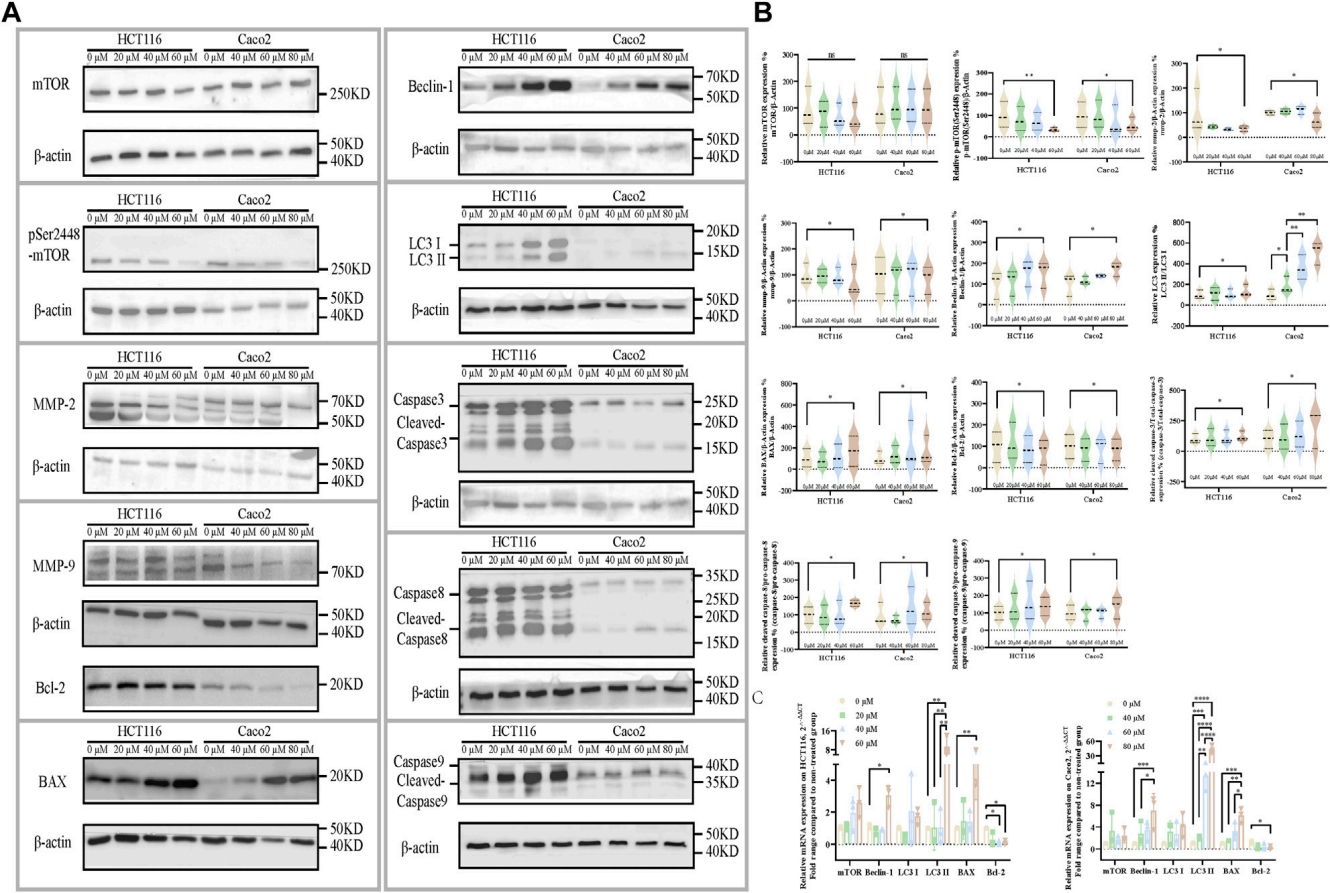
Ezetimibe downregulates the mTOR signaling, companied with a downstream invasion inhibition, apoptosis and autophagy activation. **(A)** Protein expression of mTOR, mTOR^pSer1448^, MMP-2, MMP-9, BAX, Bcl-2, Beclin-1, LC3, caspase-3, caspase-8, caspase-9 and β-actin in HCT116 and Caco2 cells under gradient ezetimibe treatment. **(B)** Statistical analysis for the protein expression level of mTOR/β-actin, mTOR^pSer1448^/β-actin, MMP-2/β-actin, MMP-9/β-actin, Beclin-1/β-actin, LC3 II/LC3 I, BAX/β-actin, Bcl-2/β-actin, cleaved caspase-3/total caspase-3, cleaved caspase-8/pro-caspase-8 and cleaved caspase-9/pro-caspase-9 in HCT116 and Caco2 cells under gradient ezetimibe treatment. **(C)** Statistical analysis for the relative mRNA expression of mTOR, Beclin-1, LC3 I, LC3 II, BAX and Bcl-2 of gradient ezetimibe treated HCT116 and Caco2 cells. *p < 0.05, **p < 0.01, ***p < 0.001, ****p < 0.0001.

Molecular level evaluation revealed the apoptosis-inducing, autophagy-activating, and invasion-inhibiting effects of ezetimibe on CRC cells through the downregulation of phosphorylated mTOR. mTOR^pSer2448^ levels were found to decrease in HCT116 cells and Caco2 cells (Figures 4A, B) following treatment with effective ezetimibe concentrations; in addition we found no significant differences in mTOR on mRNA and protein expression levels between the two cell lines (Figures 4A–C). Cysteinyl aspartate-specific proteinases were activated 24 h following effective ezetimibe treatment. The protein levels of the activated cleaved fragments of caspase-8 and caspase-9, which are the cascade initiators of caspase cell death, increased in CRC cells under ezetimibe treatment, as well as activated caspase-3, the apoptotic executioner (Figures 4A, B). The expression levels of BAX and Bcl-2, which are regulators of mitochondrial outer membrane permeability (MOMP), were also found to be significantly different between the ezetimibe-treated and control groups. The expression levels of pro-apoptotic BAX were found to increase with increase in ezetimibe dose on both protein and mRNA level; And the expression levels of Bcl-2, which has anti-apoptotic effects, exhibited an inverse trend to those of BAX in HCT116 cells and Caco2 cells (Figures 4A–C). Beclin-1, the main autophagy protein, exhibited an increasing trend in ezetimibe-treated HCT116 and Caco2 cells on both protein and mRNA level (Figures 4A–C). The expression levels of the autophagosome membrane signature protein, LC3II, significantly increased at effective ezetimibe concentrations in HCT116 cells and Caco2 cells (Figures 4A–C). The protein expression levels of MMP-2 and MMP-9 (Figures 4A, B), which are proteins that indicate tumor invasiveness, were found to decrease in ezetimibe-treated groups.

**FIGURE 5:**
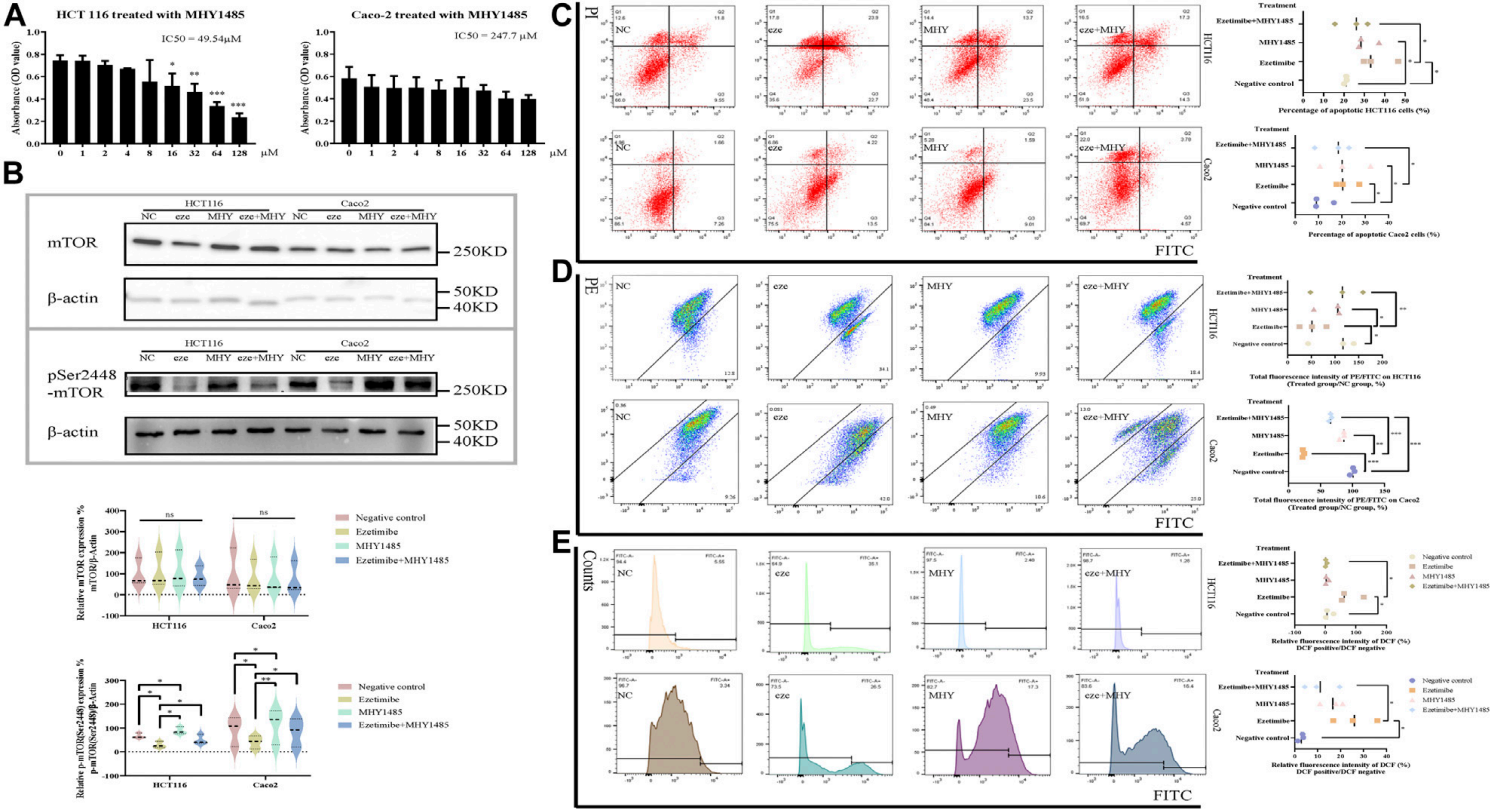
CRC cell apoptosis and mitochondrial dysfunction can be partly rescued by mTOR activator MHY1485. **(A)** Effects of MHY1485 on the viability of HCT116 and Caco2 cells at 48 h detected by CCK-8 assay. **(B)** Protein expression of mTOR and mTOR^pSer1448^ in the groups of negative control, ezetimibe, MHY1485 and ezetimibe + MHY1485 for HCT116 and Caco2 cells. **(C)** Apoptosis of HCT116 and Caco2 cells for groups of negative control, ezetimibe, MHY1485 and ezetimibe + MHY1485 with Annexin V-FITC/PI staining flow cytometry. **(D)** Fluorescence intensity of JC-1 for groups of negative control, ezetimibe, MHY1485 and ezetimibe + MHY1485 on HCT116 and Caco2 cells with flow cytometry. **(E)** Fluorescence intensity of DCF for groups of negative control, ezetimibe, MHY1485 and ezetimibe + MHY1485 on HCT116 and Caco2 cells with flow cytometry. *p < 0.05, **p < 0.01, ***p < 0.001, ****p < 0.0001.

mTOR activation reverses the ezetimibe-induced cell death phenotype, characterized mitochondrial dysfunction. To calculate the IC_50_ and choose a proper working concentration for MHY1485, a potent cell-permeable mTOR activator, for administration in HCT116 and Caco2 cells, a CCK8 assay was performed (Figure 5A). MHY1485 concentrations of 10 μM and 20 μM were chosen for HCT116 and Caco2 cells based on the calculated IC_50_ and the findings of previous studies (Liu et al., 2020; Wu et al., 2020; Yang et al., 2020). For both cell lines, mTOR^pSer2448^ expression was found to significantly decrease in the effective ezetimibe-treated group as compared to the negative control group; there was also a difference in mTOR^pSer2448^ expression between the ezetimibe- and MHY1485-treated groups, as well as between the negative control and MHY1485-treated groups (Figure 5B). mTOR^pSer2448^ expression was partially activated by MHY1485 in the ezetimibe + MHY1485-treated group compared to ezetimibe-treated group (Figure 5B). In addition, there was no difference in mTOR protein expression levels between the negative control, ezetimibe-treated, MHY1485-treated, and ezetimibe + MHY1485-treated groups (Figure 5B). The reversal of ezetimibe-induced phosphorylated mTOR inhibition by MHY1485 was accompanied by a decrease in apoptotic cell counts and an improvement in mitochondrial damage. Flow cytometric detection revealed a homodromous difference between Caco2 and HCT116 cells (Figure 5C). The ezetimibe-induced decrease in mitochondrial membrane potential was partially improved by MHY1485 in both HCT116 cells and Caco2 cells (Figure 5D). In addition, intracellular ROS accumulation in CRC cells was also partially decreased by the mTOR activator (Figure 5E).

### Ezetimibe exerts anti-CRC effects

**FIGURE 6:**
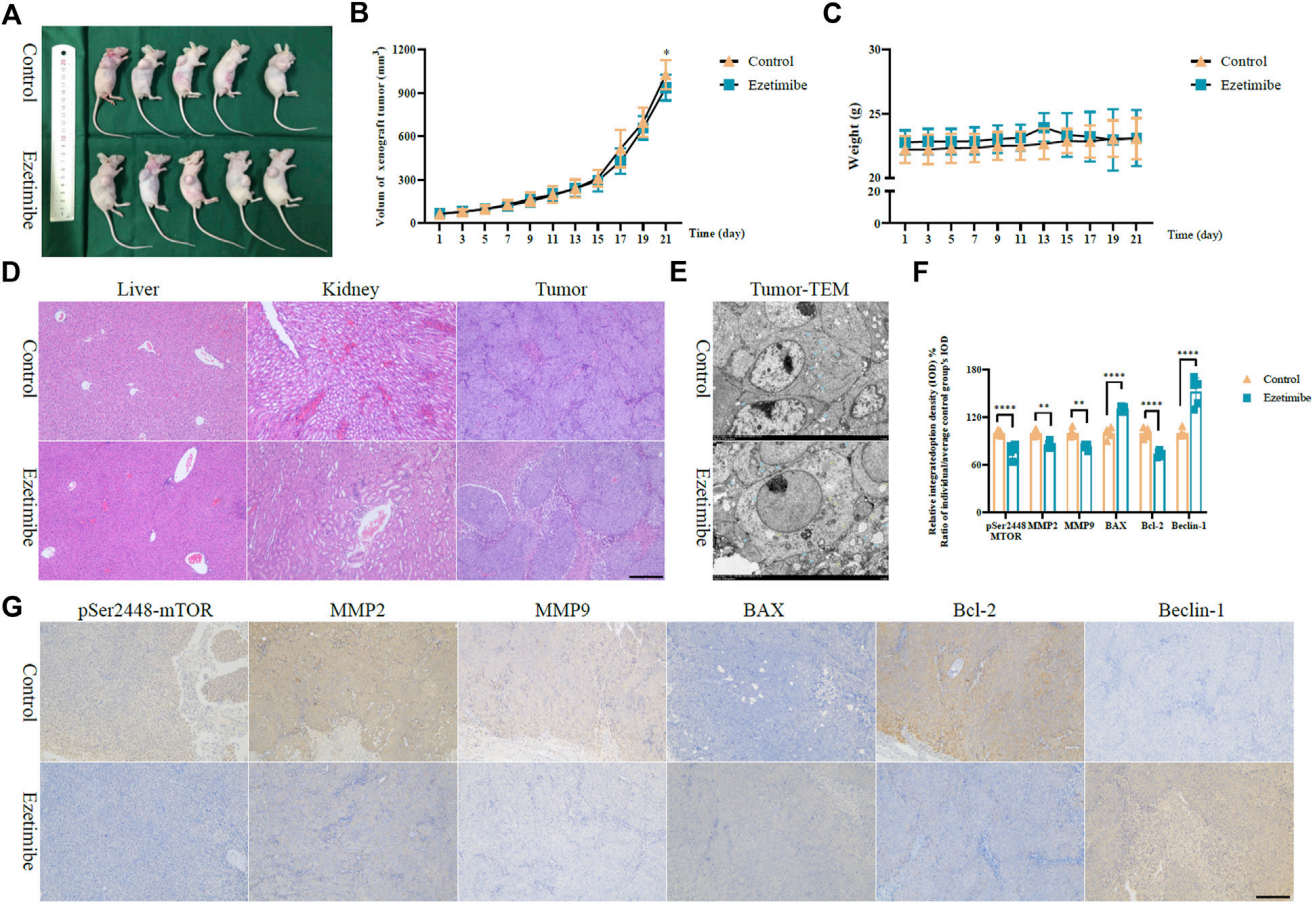
Ezetimibe functions an anti-colorectal cancer role in xenograft mice model. **(A)** Subcutaneous HCT116 xenograft mice model of control and ezetimibe treated groups. **(B)** The volum of subcutaneous tumor in groups of control and ezetimibe. **(C)** The mouse weights of control and ezetimibe treated group. **(D)** H&E staining measurement for liver and kidney in groups of control and ezetimibe. **(E)** Transmission electron microscopic observations for groups of control and ezetimibe. **(F)** Statistical analysis for the immunohistochemistry of mTOR^pSer1448^, MMP-2, MMP-9, BAX, Bcl-2, Beclin-1 in groups of control and ezetimibe. **(G)** Immunohistochemical staining for mTOR^pSer1448^, MMP-2, MMP-9, BAX, Bcl-2, Beclin-1 in groups of control and ezetimibe. *p < 0.05, **p < 0.01, ****p < 0.0001.

The findings of the experiments carried out on the xenograft tumor mouse model demonstrated the anti-CRC effects of ezetimibe. The tumor volume of HCT116 cells subcutaneously implanted into nude mice decreased following treatment with ezetimibe for 3 weeks; in addition, no significant difference in mouse weight was observed between the control group and the ezetimibe-treated group (Figures 6A–C). The H&E staining analysis did not reveal significant morphological changes in mouse livers and kidneys between the control group and the ezetimibe-treated group (Figure 6D); this indicated the low toxicity of ezetimibe on key metabolic organs. The TEM analysis revealed morphological changes such as mitochondrial spine reduction and increase in autophagosome counts in the ezetimibe-treated group as compared to the control group (Figure 6E). Comparing protein levels between the control and ezetimibe-treated groups through immunohistochemical staining showed results similar to those of the cytological experiments, including apoptosis induction, autophagy activation, and the reduction of invasiveness. There was a downregulation in MMP-2, MMP-9, and Bcl-2 expression, as well as a decrease in mTOR^pSer2448^ expression, in the ezetimibe-treated group as compared to the control group; In addition, the expression levels of BAX and Beclin-1 were upregulated in the ezetimibe-treated group (Figures 6F, G).

## Discussion

CRC, which is the fourth most common type of cancer, causes approximately 394,000 deaths worldwide annually (Boyle and Langman, 2000). In the last decades, its incidence in younger individuals has significantly increased (Stoffel and Murphy, 2020). Epidemiological analyses on life style habits have shown that a westernized dietary pattern, characterized by high fat intake and low fiber ingestion, could be a contributing factor to CRC morbidity (Boyle and Langman, 2000; Stoffel and Murphy, 2020; Vernia et al., 2021). With the inadequacies of current therapeutic approaches, overall survival is still poor in advanced stage CRC patients (Boyle and Langman, 2000). Previous studies pointed out the antitumor potential of ezetimibe against several cancers involving urinary system, digestive system and genital system (Gu et al., 2022), with clinical trials not provided evidence for adverse impacts of ezetimibe on cancer risk (Peto et al., 2008; Kobbero Lauridsen et al., 2017). Based on the approach of reducing lipid absorption in the small intestines, we evaluated the potential effects of ezetimibe against CRC for the development of a novel adjuvant therapeutic strategy.

Cellular experiments showed that ezetimibe decrease proliferation, inhibit invasion, and stimulate apoptosis in CRC cell lines (Figures 1, 2). IC_50_ was used to evaluate CRC cell viability in this study (Sebaugh, 2011). HCT116 cell viability was found to decrease by half when ezetimibe concentrations reached 73.71 μM and 56.85 μM at 24 and 48 h, respectively (Figure 1B). A similar trend was observed in the Caco2 cell line, with IC_50_s of 79.27 μM and 44.33 μM at 24 and 48 h, respectively (Figure 1C). The clone-formation capacity of CRC cells, which represents the proliferative viability of the cells (Dobson et al., 2020), also decreased following treatment with ezetimibe (Figure 1D). CRC cell invasiveness was found to significantly decrease following ezetimibe treatment in a dose- and time-dependent, as determined through the wound healing assay (Figures 1E, F). In addition, CRC cell apoptosis increased following treatment with effective ezetimibe concentrations as determined through the fluorescence microscopic and flow cytometric assays (Figures 2D, E); these findings indicated the toxic effects of ezetimibe against CRC.

Energy metabolism in cancer cells is more complex than simple glycolysis, as initially reported, and involves the use of a wide variety of substrates (Ferro et al., 2020). To satisfy the energy demands of cancer cells, mitochondria have to be trafficked from host immune cells, highlighting the important role played by mitochondria as powerpacks in cancer cells (Ferro et al., 2020; Saha et al., 2022). The ATP Binding Cassette transporter (ABCC) subfamily of ABCA1 and ABCC3, as potential targets for ezetimibe (Figure 3), reported of intervening the drug resistance in cancers (Wang and Smith, 2014; Grube et al., 2018; Belisario et al., 2020; Ramírez-Cosmes et al., 2021; Gao et al., 2022), thus providing evidence for the possibility of using ezetimibe in CRC adjuvant therapy. We found ezetimibe-induced CRC cell death to be associated with mitochondrial dysfunction. The JC-1 fluorochrome, a cyanine dye widely used to distinguish energized mitochondria from de-energized ones (Perelman et al., 2012), was used to measure CRC △Ψm in this study. The microscopic and flow cytometric assays consistently showed an increase in the proportion of JC-1 monomers with respect to JC-1 aggregates in CRC cells with increase in ezetimibe concentration (Figures 2C–F), indicating a decrease in △Ψm in a dose-dependent manner. Excessive accumulation of intracellular ROS can induce DNA damage and finally, cell death (Filomeni et al., 2015). Following treatment with cell lethal concentrations of ezetimibe, there was a significant accumulation of intracellular ROS in both HCT116 and Caco2 cells (Figures 2B, G). The findings of these experiments revealed that mitochondrial damage occurred during ezetimibe-induced CRC cell death, indicating that an energy metabolism-related mechanism underlies the pharmacological action of ezetimibe against CRC.

The Comparative Toxicogenomics Database (Davis et al., 2019) and Drugbank online (Law et al., 2014) were used to determine the possible signaling targets of ezetimibe in CRC cells, and 29 ezetimibe-targeted genes were identified, mainly clustered into groups of glycometabolism and lipometabolism (Figures 3A, B; Table 2). The GEPIA-based analysis (Tang et al., 2017) showed that eight out of 29 genes i.e., *ABCA1*, *ABCC3*, *HMGCR*, *LDLR*, *PCSK9*, *PGR*, *PLA2G7*, and *SCARB1*, can significantly discriminate between CRC tumors and normal tissues (Figure 3C; Table 2). Among the eight targeted genes, *HMGCR*, *LDLR*, *PCSK9*, *PLA2G7*, and *SCARB1* were overexpressed in CRC tumor tissues as compared to normal tissues (Figures 3F–K), while *ABCA1*, *ABCC3*, and *PGR* exhibited low expression levels in CRC tumor tissues (Figures 3D–I). The expression levels of *PGR* vary between different CRC stages and are significantly correlated with CRC patient overall survival (Figure 3I), as its expression levels have been shown to be negatively correlated with CRC prognosis (Zhang et al., 2021). Similarly, in previous studies, *ABCC3* differential expression in different CRC stages (Figure 3E) was found not to be significantly correlated with CRC prognosis (Kim et al., 2020). *UGT2B15* and *UGT2B7*, which are non-CRC tumor-associated genes that were identified in the normal expression group, were found to be correlated with overall survival in CRC patients (Table 2). In this study, using the GEPIA database, we analyzed the correlation between the 29 targeted genes and GLS, HIF1-α, mTOR, TNF, and p53, which are recognized as key molecules involved in cellular metabolism (Table 2). Of the eight CRC-related genes, TNF was found to be significantly correlated with two of them i.e., *ABCA1* and *PLA2G7* (Table 2). In addition, of these eight genes, GLS, a key enzyme involved in glutamine metabolism, which plays an important role in cancer metabolism (Mates et al., 2019; Masisi et al., 2020), was associated with *ABCA1*, *PLA2G7*, and *SCARB1* (Table 2). p53, an important antitumor transcription factor in CRC cells (Liebl and Hofmann, 2021), was found to be associated with four of the CRC-related expression genes i.e., *ABCA1*, *PCSK9*, *PGR*, and *SCARB1* (Table 2). A total of 6 and seven CRC-related expression genes were found to be significantly correlated with mTOR and HIF1-α, respectively, in the mTOR signaling pathway (Table 2), indicating that mTOR signaling is an important part of the mechanisms underlying the effects of ezetimibe against CRC. Low density lipoprotein receptor (LDLR), which is significantly correlated to mTOR according to the bioinformatic analysis (Table 2), mediates the hepatic endocytosis to consume Niemann-Pick C1-Like 1 (NPC1L1) mediated lipid absorption (Gu et al., 2022; Liao et al., 2022; Zhang et al., 2022). AS ezetimibe exerts its suppression on cholesterol absorption with blockage to NPC1L1, it may be potential regulator of mTOR signal for the lipid homeostasis in CRC progression. Proprotein convertase subtilisin/kexin type 9 (PCSK9) and HMG-CoA reductase (HMGCR), both significantly high expressed in CRC tumor as ezetimibe potential targets (Figure 3), also confirmed of tight correlation to mTOR (Table 2). Studies have also reported that the PCSK9 inhibitor and HMGCR suppressor statins possess anti-CRC effects (Navarese et al., 2015; Gu et al., 2022; Shailes et al., 2022), which further stand for the potential pivotal role of mTOR signal mediation by ezetimibe in CRC. The ezetimibe targeted phospholipase A2 group VII (PLA2G7) has also been found to have tight correlation with cancer cachexia (Morigny et al., 2021) and put forward as therapeutic target for prostate cancer (Vainio et al., 2011), supporting the possibility of ezetimibe used in clinical therapy in advanced cancer stages.

Western blotting and the quantitative real-time polymerase chain reaction (qRT-PCR) assay carried out in our study revealed that ezetimibe attenuated CRC invasiveness, and induced CRC apoptosis and autophagy, which were accompanied by a decrease in mTOR phosphorylation at the molecular level. The mTOR^pSer2448^ protein, which is recognized as a key indicator of mTOR signaling activation, was found to be negatively correlated with disease prognosis in advanced stage CRC patients (Vainio et al., 2011; Muller et al., 2013; Navarese et al., 2015; Mates et al., 2019; Kim et al., 2020; Masisi et al., 2020; Zhang et al., 2020; Liebl and Hofmann, 2021; Morigny et al., 2021; Liao et al., 2022; Shailes et al., 2022; Zhang et al., 2022), its expression levels significantly decreased in both HCT116 and Caco2 cells following effective ezetimibe treatment (Figures 4A, B); however, mTOR transcription and expression levels were not significantly different between the ezetimibe-treated and control groups (Figures 4A–C). MMPs play crucial roles in tumorigenesis as they promote angiogenesis, invasiveness, and immune evasion in a wide variety of cancers (Vainio et al., 2011; Muller et al., 2013; Navarese et al., 2015; Gobin et al., 2019; Masisi et al., 2020; Zhang et al., 2020; Liebl and Hofmann, 2021; Lodge et al., 2021; Morigny et al., 2021; Liao et al., 2022; Shailes et al., 2022; Zhang et al., 2022). Ezetimibe induced MMP-2 and MMP-9 downregulation in HCT116 and Caco2 cells (Figures 4A, B); this finding, together with the decreased wounding healing capacity found in the scratch assay, demonstrated the inhibitory effects of ezetimibe against CRC invasiveness. Ezetimibe induced CRC cell apoptosis and autophagy at effective concentrations. In this study, we observed an increase and a decrease in both BAX and Bcl-2 mRNA and protein levels, respectively, in the CRC cell lines; this finding, together with the caspase cascade activation observed (Figures 4A–C), provided evidence for our point of the CRC cell apoptosis promotion effects of ezetimibe. The expression levels of Beclin-1, the pre-autophagosomal structure positive correlated protein (Tran et al., 2021), significantly increased under treatment with increasing ezetimibe concentrations (Figures 4A–C). In addition, the expression levels of the key autolysosome membrane formation protein, LC3 II, were also found to significantly increase in ezetimibe-treated HCT116 and Caco2 cells in a dose-dependent manner (Figures 4A–C).

The apoptosis and mitochondrial dysfunction phenotype observed in ezetimibe-treated CRC cells was partially reversed by the mTOR activator, MHY1485 (Figure 5), indicating that ezetimibe induces cell death and mitochondrial dysfunction in CRC cells through the mTOR signaling pathway. The levels of mTOR^pSer2448^ proteins significantly decreased in the ezetimibe-treated group as compared to the negative control group; these levels were partially upregulated following the addition of MHY1485 to the CRC cells. The proportion of apoptotic HCT116 and Caco2 cells decreased in the ezetimibe + MHY1485-treated group as compared to the ezetimibe-treated group, and this was accompanied by homogeneous △Ψm recovery and a reduction in intracellular ROS levels.

The anti-CRC effects of ezetimibe, which involve the promotion of CRC cell death without damage to vital organs involved in drug metabolism, were confirmed using a xenograft tumor mouse model (Figure 6). Ezetimibe significantly decreased the volumes of xenograft tumors in nude model mice subjected to treatment as compared to control mice; however, there was no significant difference in mouse weight between the two groups (Figures 6A–C). No significant morphological changes were observed in the livers and kidneys of mice in the control and ezetimibe-treated groups as determined through the H&E staining analysis (Figure 6D); however, visible mitochondrial morphological damage, with cytophagosome development, was observed by TEM in the xenograft tumor tissues of ezetimibe-treated mice (Figure 6E). HCT116 xenograft mouse tumor immunohistochemical staining showed an increase in the expression levels of apoptosis/autophagy-related proteins and a decrease in the expression levels of invasiveness-related proteins, considering the downregulation of mTOR^pSer2448^ expression in the ezetimibe-treated group (Figures 6F, G). *In vivo* findings support our hypothesis that ezetimibe induces CRC cell death *via* mTOR signaling-dependent mitochondrial dysfunction.

Despite the demonstrated potential anti-CRC effects of ezetimibe, our study still has some limitations. First, the in-depth mechanisms of ezetimibe-induced mitochondrial dysfunction, as well as correlations to the tumor microenvironment in CRC, still need to be investigated. More evidence needs to be gathered on the clinical application of ezetimibe in the treatment of CRC before it be taken from the bench to the bedside for CRC treatment.

## Conclusion

**FIGURE 7:**
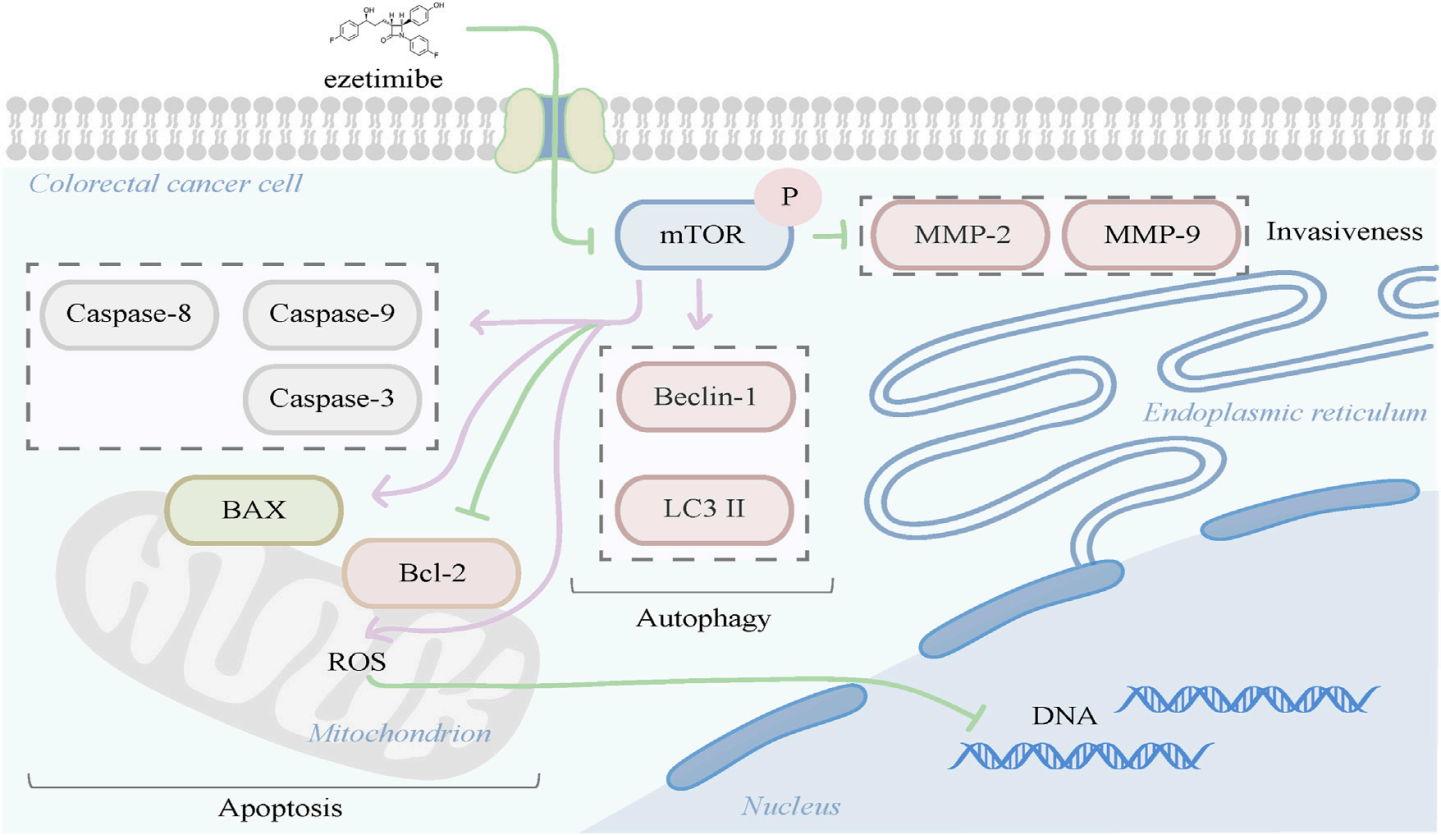
Ezetimibe induced colorectal cell death were found to be correlated with mTOR signaling-dependent mitochondrial dysfunction in apoptosis promotion, meanwhile presented effects of autophagy activation and invasiveness reduction.

We demonstrated *in vitro* and *in vivo* that ezetimibe elicits its anti-CRC effects by inhibiting cell proliferation and invasion, and promoting apoptosis and autophagy. In addition, ezetimibe-induced apoptosis and autophagy were found to be correlated with mTOR signaling-dependent mitochondrial dysfunction (Figure 7). The findings of this study highlight the potential value of ezetimibe for use in CRC adjuvant therapy and for optimized selections in individual therapeutic schedules in future.

## Data Availability

The original contributions presented in the study are included in the article/Supplementary Material; further inquiries can be directed to the corresponding authors.

## Supplementary material

Supplementary Figure S1 Ezetimibe suppresses the Caco2 cell migration in low doses. **(A)** The wound healing capacity for Caco2 under gradient ezetimibe treatment at 0, 24, and 48 h. Scale bar, 100 μM. **(B)** Statistical analysis for the wound area of Caco2 under gradient ezetimibe treatment at 0, 24 and 48 h. **p* < 0.05, ****p* < 0.001.

Supplementary Figure S2 Ezetimibe downregulates the MMP2, MMP9 and Bcl-2, companied with caspase-8, caspased-9 activation. **(A)** Protein expression of MMP-2, MMP-9, Bcl-2, caspase-8, caspase-9 and β-actin in HCT116 and Caco2 cells under gradient ezetimibe treatment. **(B)** Statistical analysis for the protein expression level of MMP-2/β-actin, MMP-9/β-actin, Bcl-2/β-actin, cleaved caspase-8/pro-caspase-8 and cleaved caspase-9/pro-caspase-9 in HCT116 and Caco2 cells under gradient ezetimibe treatment. **p* < 0.05, ***p* < 0.01.
