# Supplementary figures and images for: Promotion of colorectal cancer cell death by ezetimibe via mTOR signaling-dependent mitochondrial dysfunction
Source: Front Pharmacol. 2023 Feb 7;14:1081980. doi: 10.3389/fphar.2023.1081980 (PMC9946110; doi:10.3389/fphar.2023.1081980)

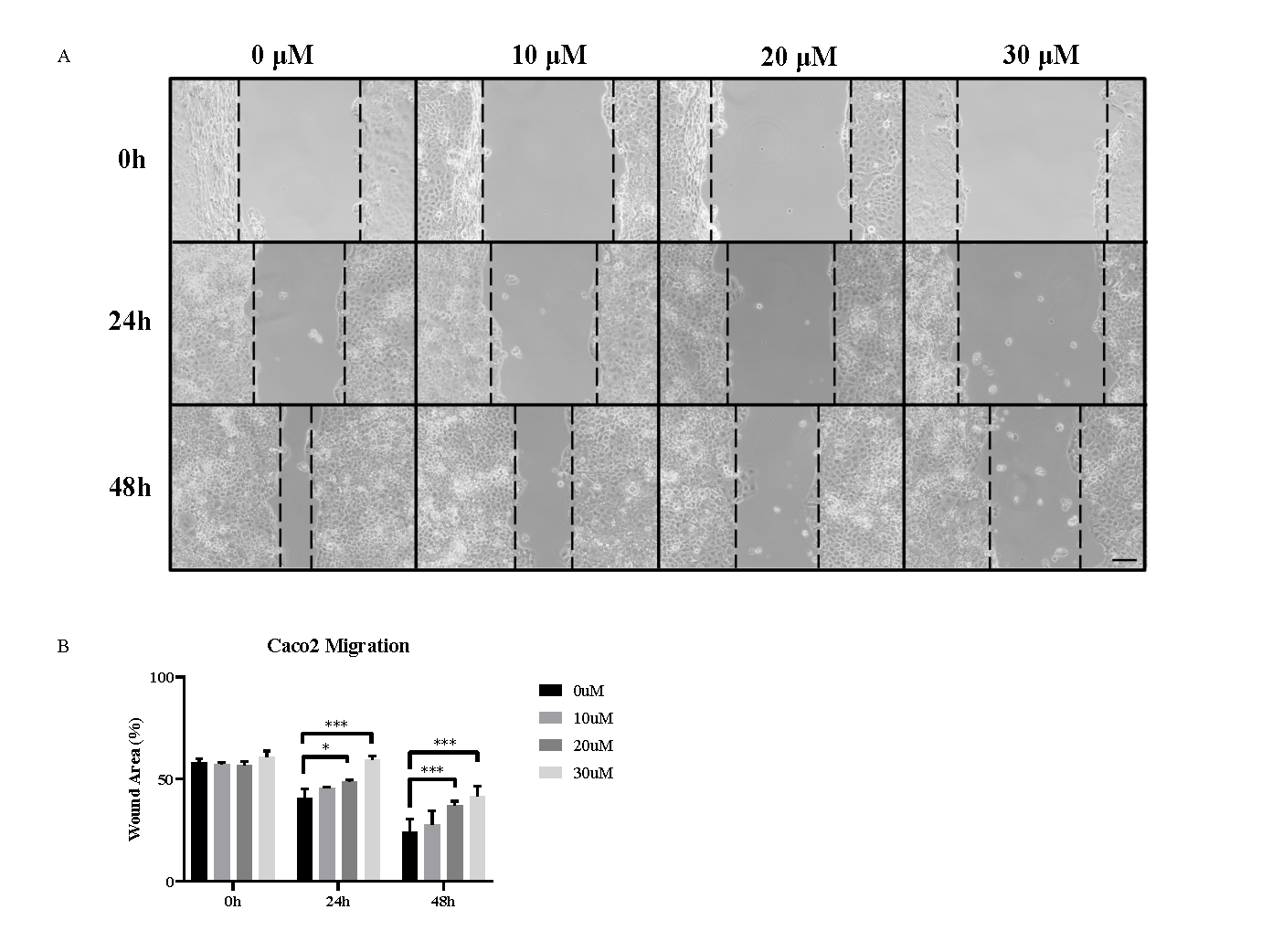

Supplement: Supplementary file 1 [file Image1.tiff]

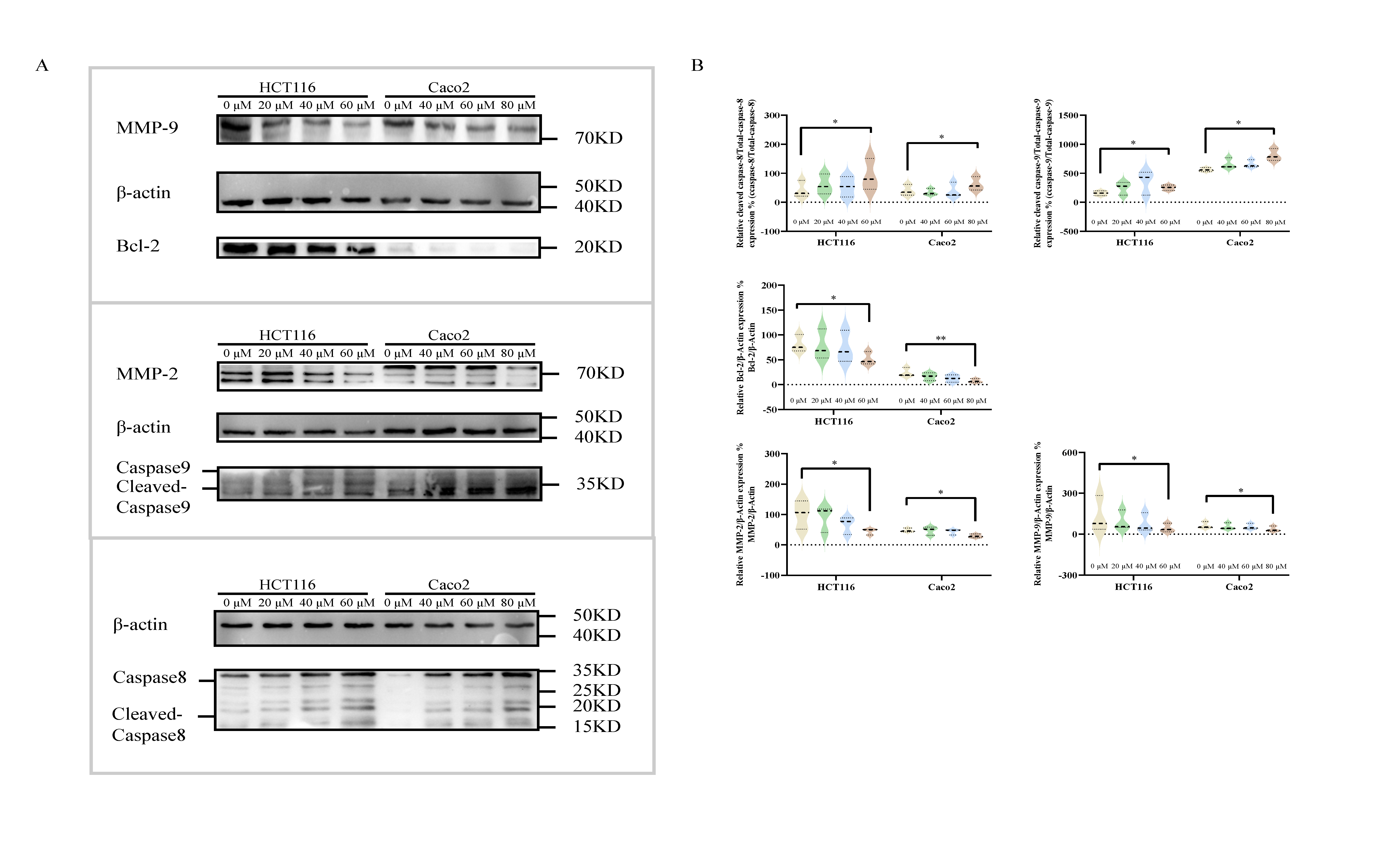

Supplement: Supplementary file 2 [file Image2.tif]
